# Research Progress on the Role of ABC Transporters in the Drug Resistance Mechanism of Intractable Epilepsy

**DOI:** 10.1155/2015/194541

**Published:** 2015-09-27

**Authors:** Jie Xiong, Ding-an Mao, Li-qun Liu

**Affiliations:** Division of Pediatric Neurology, Children's Medical Center, The Second Xiangya Hospital of Central South University, Changsha, Hunan 410011, China

## Abstract

The pathogenesis of intractable epilepsy is not fully clear. In recent years, both animal and clinical trials have shown that the expression of ATP-binding cassette (ABC) transporters is increased in patients with intractable epilepsy; additionally, epileptic seizures can lead to an increase in the number of sites that express ABC transporters. These findings suggest that ABC transporters play an important role in the drug resistance mechanism of epilepsy. ABC transporters can perform the funcions of a drug efflux pump, which can reduce the effective drug concentration at epilepsy lesions by reducing the permeability of the blood brain barrier to antiepileptic drugs, thus causing resistance to antiepileptic drugs. Given the important role of ABC transporters in refractory epilepsy drug resistance, antiepileptic drugs that are not substrates of ABC transporters were used to obtain ABC transporter inhibitors with strong specificity, high safety, and few side effects, making them suitable for long-term use; therefore, these drugs can be used for future clinical treatment of intractable epilepsy.

## 1. Introduction

Epilepsy is a group of chronic brain diseases characterized by the transient dysfunction of the central nervous system that is induced by abnormal discharge and threatens human health. Finding a treatment for epilepsy has been a priority in drug development. There are more than 20 types of antiepileptic drugs (AEDs) at present. Approximately 2/3 of epilepsy patients can achieve satisfactory results through antiepileptic drug treatment, but approximately 1/3 of epilepsy patients cannot control their symptoms using multiple AED treatments and may develop intractable epilepsy (IE). Although there is no consensus regarding the definition of IE, this condition is generally diagnosed based on a lack of change in the therapeutic index such that the number of episodes does not decrease or increase after sequential or combined application of at least two types of antiepileptic drugs with a sufficient or tolerable dose for a sufficiently long period.

The resistance mechanism of IE remains unclear, and two main features of this resistance have attracted attention: (1) excessive expression of a transporter that can pump the drugs out of the brain cells, which causes a reduction in the local concentration (at epilepsy lesions) of AEDs that pass through the blood brain barrier (BBB), and (2) resistance due to changes in drug targets, such as reduced or changed sodium channels or GABA receptors. Clinical studies have identified epilepsy patients with drug resistance against a specific type of AED; most of these patients could develop drug resistance if they changed to an AED with a different action mechanism, and symptoms were controlled in only 10% of the patients [1]. Therefore, IE may have a nonspecific resistance mechanism, leading to both a low concentration of various drugs in the epileptic foci of brain tissue and drug resistance, and this mechanism may have very little to do with the targets of AED activity [2]. Excessive expression of some efflux transporters in the microvascular endothelial cells of epilepsy lesions may cause the drug resistance in IE patients. Rambeck et al. [3] identified 22 cases of patients with drug-resistant epilepsy and compared the concentrations of AEDs in the extracellular fluid of the cortex, brain tissue, subarachnoid cerebrospinal fluid, and serum through intraoperative micropermeability analysis. The authors found that the concentrations of carbamazepine and lamotrigine in the extracellular fluid of the cortex were significantly lower than that in the cerebrospinal fluid (CSF), and the drug concentration in the target brain tissue differed substantially from that in the CSF and serum. The results verified the theory of a multidrug transport protein in the brain tissues of IE patients. Multidrug transport proteins in brain tissue can pump AEDs from brain tissues, leading to drug resistance at the targeted site due to a decreased drug concentration. This hypothesis is supported by substantial evidence. Of the numerous drug transporters, the family of ABC transporters has attracted much attention. Studies have confirmed that the overexpression of ABC transporters has played an important role in multidrug resistance. Here, the role and importance of ABC transporters in the drug resistance of IE are reviewed.

## 2. ABC Transporters and Their Functions

ABC transporters received their name because they contain one or two ATP-binding cassettes (ABCs). ABC transporters are a type of transmembrane transporter, and they have two ATP-binding cassettes and two transmembrane domains. The half-transporter structure (an ABC and a transmembrane domain) of ABCG2 (BCRP) is only functional in a dimer. The ABC transporter mainly transports a combined substrate from the inside to the outside of cells using the energy produced by ATP hydrolysis. ABC transporters have various substrates, such as poisons and drugs, and they provide an important mechanism for protecting brain nerve function. The family of ABC transporters has been divided into seven subfamilies (ABCA, ABCB, ABCC, ABCD, ABCE, ABCF, and ABCG) according to the genetic structure and the arrangement of amino acids [4]. The most studied transmembrane proteins associated with multidrug resistance include P-glycoprotein (P-gp, ABCB1), multidrug resistance-associated proteins (MRPs, ABCC), and breast cancer resistance protein (BCRP, ABCG2).

### 2.1. P-gp (MDR1/ABCB1)

P-gp was first isolated from cancer cells with multidrug resistance (MDR), and this protein is also known as MDR1. This single-stranded transmembrane glycoprotein is composed of more than 1280 amino acid residues and is coded by the ABCB1 gene in human chromosomal region 7q21.1. This protein has a single nucleotide polymorphism (SNP) located at 3435 (C to T) of exon 26. P-gp is a type of efflux transporter for drugs and has been extensively researched in recent years. P-gp is mainly distributed in organs associated with absorption, metabolism, and excretion, such as the liver and kidney and organs in the digestive tract, as well as in some locations that provide protective barriers (BBB, BTB, and placental barrier) in vivo. In the human BBB, P-gp is located on the apical side of brain capillaries and in the astrocyte foot processes surrounding the capillaries, which form part of the BBB [5]. In physiological conditions, P-gp, as a type of biological barrier, can prevent the entrance of foreign substances and toxins from the extracellular fluid and maintain the stability of the internal environment. P-gp can defend and protect organs and can restrict the entry of drugs or poisons into the brain. In pathological conditions, such as during its overexpression on the surface of cancer cells, P-gp is involved in multidrug resistance [6]. In epilepsy, changes in the brain internal environment can temporarily destroy the integrity of the BBB; when the expression of P-gp is increased in the BBB, the function of the BBB can be maintained to prevent the entry of harmful substances into the brain [7]. P-gp has numerous substrates, most of which are lipophilic, including antiepileptic drugs (phenobarbital, carbamazepine, phenytoin, and lamotrigine), antitumor drugs, immunosuppressants, adrenocortical hormone, analgesics, cytokines, histamines, calcium channel blockers, cardiac glycosides, antidepressant drugs, and antibiotics (Table 1) [8]. There is a competitive relationship among substrates. When two substrates competitively bind to P-gp, the one with higher affinity is easily pumped out of the cells, whereas the one with lower affinity accumulates in the cells. Therefore, a P-gp substrate with high affinity can be used as a P-gp inhibitor. Many substances might be both the substrates and inhibitors of P-gp. They serve as substrates at a low concentration and inhibitors at a high concentration [9, 10].

There are dozens of P-gp inhibitors, which can be divided into several categories: calcium channel blockers, such as verapamil and nifedipine; calmodulin antagonists, such as trifluoperazine and chlorpromazine; cyclosporines, such as cyclosporine A; hormones and antihormone compounds, such as progesterone; protein kinase inhibitors; isoquinoline-type alkaloids, such as berberine; and others.

### 2.2. MRP (ABCC)

The protein coded by ABCC is also known as multidrug resistance-associated protein (MRP). Nine subtypes of MRP (MRP1-MRP9) associated with drug transport have been found [11]. The most studied subtype is MRP1.

MRP1 is the subtype of the MRP family that is most closely associated with multidrug resistance, and it shares 15% of its amino acid sequence with P-gp. Its gene, located at chromosomal region 16p13.1, encodes 1531 amino acids. MRP1 is commonly distributed in normal human tissues, especially in kidney, lung, and testicular tissues. Unlike P-gp, MRP1 in the central nervous system is mainly distributed in the choroid plexus epithelium and ependymal epithelium cells, which are involved in preserving the blood cerebrospinal fluid barrier (BCB) to prevent the entry of harmful substances or drugs into brain tissue. MRP1 is specific organic anion transporter. The range of MRP-mediated efflux drug is relatively narrow: basically, antitumor drugs and some antiepileptic drugs [12]. The substrates of P-gp and MRP1 overlap; in particular, a variety of lipophilic drugs can be used as the substrates of one multidrug transporter, and one drug can be used as the substrate for many multidrug transporters.

Inhibitors of MRP include probenecid, sulfinpyrazone, benzbromarone, dipyridamole, quinolines, and cyclosporines.

### 2.3. BCRP (ABCG2)

The ABCG2 gene is located at chromosomal region 4q22. ABCG2 encodes a protein containing 655 amino acids. The protein expressed by ABCG2 is called breast cancer resistance protein (BCRP). Similarly to P-gp and MRP, BCRP is an ATP-dependent drug efflux transporter. However, unlike P-gp and MRP1, ABCG2 is an atypical transporter. Structurally, BCRP has only one ATP-binding cassette and one hydrophobic transmembrane domain. Therefore, BCRP is called a half ABC transporter. BCRP is located in various tissues and cells; it is highly expressed in the gastrointestinal tract, liver, kidney, and brain and is mainly located in the cell membrane. BCRP can be highly expressed in intestinal epithelial cell membranes, liver tubular membranes, cavity surfaces of cerebral microvascular endothelial cells, and membranes of renal proximal tubule cells. BCRP participates in the transmembrane transport of its substrate drugs in vivo [13]. BCRP has a wide range of substrates, such as antitumor medicines (e.g., mitoxantrone and topotecan), tyrosine kinase inhibitors (e.g., TKI, imatinib, and gefitinib), and some antibiotic and HMG-CoA inhibitors [12]. The substrates overlap partly with those of P-gp and MRP2.

Studies have shown that some antiviral drugs have inhibitory effects on BCRP. The potency of the inhibitory effects of these drugs on BCRP is in the following order: lopinavir > nelfinavir > efavirenz > saquinavir > atazanavir > amprenavir > abacavir [14, 15].

In addition to their transmembrane transport function, ABC transporters have other physiological functions. ABCB and ABCC family members also perform various roles in the immune system. A recent study showed that the inhibition of transporters may disrupt the antitumor immune response [16]. The role of signaling pathways depends on the transport of signal molecules. The MRP family can effectively discharge prostaglandin. Studies have shown that a variety of prostanoid signaling molecules (including PGE2, PGD2, and PGF2*α*) are substrates of ABC transporters (MRP1, MRP2, and MRP4) [17, 18]. In addition, ABC transporters are involved in the transport of leukotriene signal molecules and sphingosine 1-phosphate [19]. ABC transporters promote physiological functions in cells, such as survival, proliferation, and invasion. Another important discovery regarding P-gp function is its apoptosis inhibition. This discovery established an organic connection between drug resistance and apoptosis tolerance at the molecular level. Apoptosis can be divided into two types: caspase dependent and caspase independent. P-gp can delay the apoptotic cascade and protect drug-resistant cells from various forms of caspase-dependent apoptosis (e.g., the apoptosis induced by cytotoxic drugs, free radicals, radiation, and similar factors), mainly through the activation of caspase-3 and caspase-8. However, P-gp does not inhibit caspase-independent apoptosis in drug-resistant cells. Therefore, further study of the physiological functions of ABC transporters is necessary. The results of such study would not only improve the awareness of potential adverse reactions but also reveal potential new therapeutic targets and methods.

## 3. High Expression of ABC Transporters in IE Patients and Their Influence on Epilepsy Treatment

### 3.1. High Expression of ABC Transporters in IE Patients

**Figure 1:**
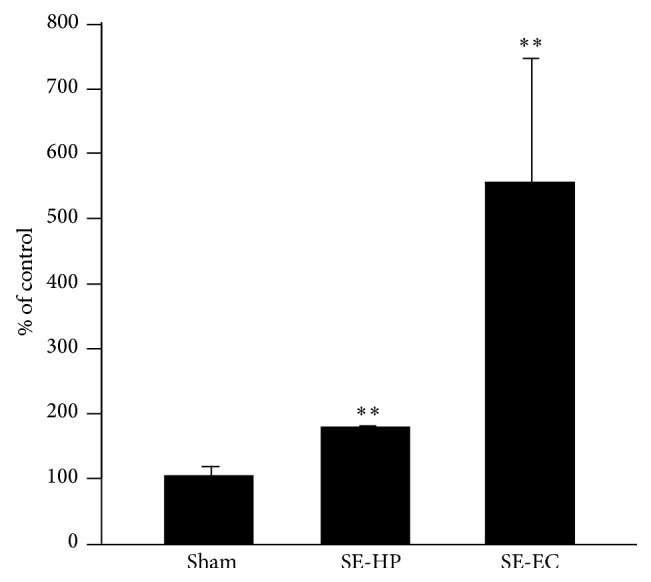
mdr1 mRNA levels in the hippocampus and entorhinal cortex of rats with spontaneous seizures. Data are the means ± SE (*n* = 5-6) of the optical density values of gel bands representing the PCR amplification products of mdr1 mRNA normalized to the corresponding *β*-actin band used in each sample as an internal standard. Values in spontaneously epileptic rats (SE) are expressed as a percentage of the control levels (sham-stimulated rats (Sham)). HP, stimulated hippocampus (1.8-fold increase); EC, contralateral entorhinal cortex (5.5-fold increase). ^∗∗^ *P* < 0.01 versus sham according to a Mann-Whitney test.

Numerous studies have shown that ABC transporters are highly expressed in IE [20]. Rizzi et al. [21] found that the MDR gene level and P-gp expression are increased by 1.8 and 5 times in the hippocampus and entorhinal cortex, respectively, of rats after 3 months of stimulation and kindling (Figure 1) [21]. Xiao et al. [22] kindled a rat model with sodium phenytoin and phenobarbital to screen the amygdaloid nucleus. Samples were taken from the drug resistance group and the effective group after 6 weeks. Immunohistochemistry was used to detect the MDR1 expression product P-gp. The results showed that, in addition to changes in the cerebellum, the expression of P-gp in brain tissues, including the amygdala, temporal lobe, frontal lobe, and parietal lobe, was obviously higher in the drug-resistant group than in the effective group, and the difference in the P-gp expression in astrocytes was significant. Sisodiya et al. [7] collected samples from epileptic patients that had a neuroepithelial tumor with embryonic developmental anomalies, limited cortical dysplasia, and hippocampal sclerosis, and they found that the MRP1 expression was obviously increased in the above intractable epilepsy lesions. Dombrowski et al. [23] isolated capillary endothelial cells from the removed temporal lobe of patients with IE. An MDR1 (ABCB1) cDNA probe was used to measure the MDR1 gene expression. The results showed that the P-gp immune-positive samples and the expression of MDR1 in the temporal lobe vascular endothelial cells of IE patients were higher than those in the control group of temporal lobe vascular endothelial cells from aneurysm resection.

Recent studies showed not only an increase in the expression level of ABC transporters but also an increase in the number of expression sites after epileptic seizures. Under normal physiological conditions, the MDR1 gene expression of the central nervous system is limited to capillary endothelial cells and the astrocytes around the capillaries. However, after epileptic seizures, the P-gp of the patients with epileptic seizures is also expressed on parenchymal astrocytes, even on the neurons, in addition to its previous expression in brain capillary endothelial cells and astrocytes around the capillaries [24] (Table 2). Normal brain neurons and astrocytes do not express MRP, but in the samples from intractable epilepsy patients after resection (e.g., limited resections for cortical dysplasia and hippocampal sclerosis), MRP can be detected in astrocyte and hypogenetic neuron membranes [23]. This expression may provide a histological explanation for the significant correlation between drug-resistant epilepsy and the high expression of ABC transporters.

### 3.2. Effects of the High Expression of ABC Transporters on IE Treatment

Increased expression of ABC transporters in the brain will enhance the function of the BBB, and AEDs are the natural substrate of ABC transporters because of their high lipid solubility [25]; thus, a high expression of ABC transporters may limit the passage of AEDs through the BBB, prevent AEDs from entering brain tissue, and reduce the concentration of antiepileptic drugs in epilepsy brain lesions [26, 27]. Therefore, even for a blood drug concentration that is within the scope of treatment, the internal and external concentrations of parenchymal cells are not sufficient to provide the treatment effect, thus leading to drug resistance. This theory has been confirmed by considerable research. Studies have analyzed the concentration of phenobarbital (PB) in the serum and cerebrospinal fluid (CSF) of epilepsy patients, and the results showed that the daily PB dose and serum PB concentration are similar between the observation group (decreased seizure frequency ≥ 50%) and the control group (decreased seizure frequency < 50%). However, the CSF PB concentration and the CSF to serum ratio of PB in the observation group were both higher than those in the control group, suggesting that the amount of PB that penetrates the BBB is lower in the control group [28]. Rizzi et al. [21] induced epileptic seizures in mouse limbic lobes. The mRNA expression of mdr1 within 3~24 h of the seizures was increased by 85%, and this increase continued until 72 h. Six hours after the seizures, the brain tissue/plasma ratio of PB was decreased by 30% and the extracellular concentration was increased by 2-fold. Compared with wild-type mice, the knockout mice (mdr^−/−^) had a hippocampal drug concentration that was increased by 46% after the injection of phenobarbital. Carbamazepine could be detected in the mouse hippocampus at 3 h after the epileptic seizures and carbamazepine treatment but could not be detected in the wild-type mice. The experiment confirmed that an epileptiform seizure can induce the expression of the mdr1 gene in mice and that P-gp can significantly affect the AED concentration in brain tissue.

Overexpression of multidrug-resistant transport protein in IE patients is not only limited to the brain. Various areas of the intestine can also produce P-gp and thus form a barrier that limits the passage of antiepilepsy drugs from the lumen to the blood and reduces the bioavailability of oral medications. Furthermore, clinical reports have shown that, in patients with intractable epilepsy with excessive MDR1 expression, the AED blood levels are always low despite continuous AED administration [29].

Wang et al. studied 49 epilepsy patients and measured the expression of P-gp in the peripheral blood using flow cytometry. Eleven patients showed positive P-gp expression, and the subsequent treatments were all ineffective. In the remaining 32 cases with negative expression, 18 showed significantly reduced mortality after adjustment of the drug doses. Thus, overexpression of peripheral P-gp is considered an objective indicator of drug-resistant epilepsy, and this marker can be used to guide clinical treatments [30].

## 4. Possible Causes and Mechanism of the High Expression of ABC Transporters in IE Patients

### 4.1. Possible Causes of the High Expression of ABC Transporters

The reason for the overexpression of ABC transporters in the lesions of IE patients is not clear. Initially, P-gp expression was believed to be induced by chronic AED treatments. To investigate this possibility, Seegers et al. [31] treated rats with phenobarbital and phenytoin sodium for 11 d. Then, immunohistochemistry was used to detect the expression of P-gp in endothelial and brain cells, including cells of the temporal lobe, where drug resistance usually appeared. In the piriform/parietal cortex and cerebellum of rats treated by phenobarbital, the P-gp expression was moderately elevated; however, no significant increase in P-gp expression was observed in other brain regions after the phenobarbital and phenytoin sodium treatment, indicating that the excessive P-gp expression in patients with IE is not caused by chronic AED treatment.

Seegers et al. [32] measured the time course of P-gp expression in the hippocampus and other brain regions in a kainate rat model with temporal lobe epilepsy. At 24 h after the epilepsy state, the P-gp expression was significantly increased in the endothelial cells of the dentate gyrus and the parenchyma cells of the CA1 and CA3 regions of the hippocampus. At 10 d after epilepsy, there were no obvious differences with the control group other than the previously mentioned increased expression. This finding suggests that the increased expression of P-gp at 24 h after epilepsy is temporary and that the overexpression of P-gp is the result of the temporary seizure.

In patients with cortical malformations, however, overexpression of multidrug transprotein is an intrinsic change rather than a temporarily induced change [33]. Lazarowski et al. [34] found that some patients with abnormal brain developments, such as tuberous sclerosis, had a high expression of MRP prior to seizure or prior to taking AEDs. The drug resistance of intractable epilepsy is also associated with MDR1 gene polymorphism. Siddiqui et al. [35] studied 315 patients with epilepsy, including 115 patients with sensitivity to drug treatment, 200 patients with drug-resistant intractable epilepsy, and another 200 normal controls, and the researchers obtained peripheral blood samples to observe the polymorphisms of the single nucleotide ABCB1-C3435T. They found that patients with drug-resistant epilepsy had the CC genotype more frequently than the TT genotype. But results from various studies indicate that this is controversial [36].

Studies showed that MRP is also expressed in Parkinson's disease and Alzheimer's disease [37, 38].

In conclusion, the cause of the high expression of ABC transporters is believed to involve a variety of factors, which may be associated with many features, such as seizures, genes, and immunity.

### 4.2. Possible Mechanism Underlying the Upregulation of ABC Transporter Expression in IE Patients

**Figure 2:**
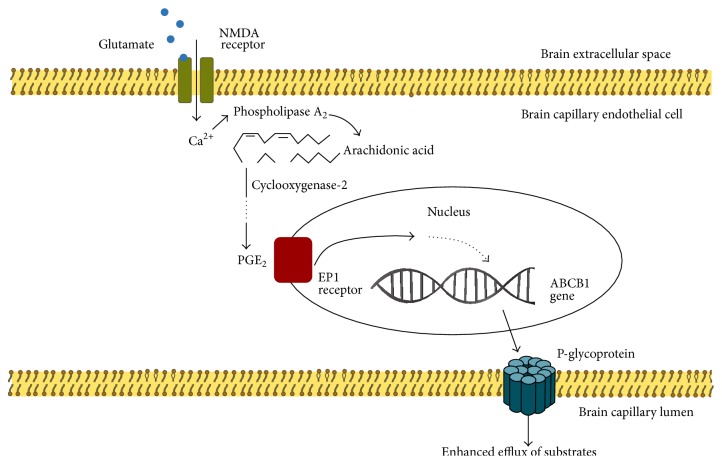
Extracellular concentrations of glutamate increase during epileptic seizures. Glutamate signaling can occur via endothelial NMDA receptors to activate an intracellular cascade that upregulates P-glycoprotein [60]. Ca^2+^ influx via the NMDA receptor is known to activate phospholipase A2, which can release arachidonic acid from the cell membrane. Therefore, Ca^2+^ might represent the link that drives the activation of arachidonic acid signaling. The inflammatory enzyme cyclooxygenase-2 was clearly demonstrated to be a key downstream effector that processes arachidonic acid [60, 61]. Prostaglandin E2, as the main end product of cyclooxygenase-2, was shown to act via the endothelial EP1 receptor [62]. The events downstream of EP1 receptors must still be identified; these events then drive the transcriptional activation of the P-glycoprotein-encoding gene.

The mechanism underlying the upregulated ABC transporter expression in IE patients is not yet clear. Multiple signal transduction pathways may be involved. Studies have confirmed [39] that excessive glutamate is associated with seizures and that the glutamic acid → N-methyl-D-aspartate (NMDA) receptor → cyclooxygenase-2 (COX-2) → prostaglandin E2 receptor → P-gp pathway is involved in the regulation of P-gp expression in epilepsy (Figure 2).

Protein kinase C (PKC) can also regulate the expression and function of P-gp. A study found that the PKC expression within epilepsy-related tissues was also increased. This effect may occur because the MDR1 gene acts through the PKC signal transduction pathway to increase MDR1 transcription and translation, thus increasing P-gp expression [40].

Recurrent epileptic seizures can induce the upregulation of the expression of a variety of proinflammatory cytokines, such as IL-1*β*, IL-6, and TNF-*α*, in brain tissue; these inflammatory factors can enhance the binding of glutamic acid to the NMDA receptor, which further aggravates seizures [41]. NF-*κ*B is a player in the regulation of inflammation. High expression of NF-*κ*B is found in neurons and glia from samples obtained during operations on epilepsy patients. The activation level of peripheral NF-*κ*B in children with epilepsy was obviously higher than that in a healthy control group; these patients were reviewed after effective antiepileptic treatment, when the activation level of peripheral NF-*κ*B was significantly decreased [42]. In in-depth studies, some scholars found that the NF-*κ*B complex can activate the transcription of the gene associated with the MDR1 promoter. The main mechanism occurs through transcriptional regulation loci on the NF-*κ*B subunit that binds with p65 in the MDR1 gene promoter region [43]. When a seizure induces inflammation, a variety of inflammatory factors can act on MDR1 through NF-*κ*B and P-gp expression is upregulated; thus, the inflammatory response is associated with drug-resistant epilepsy. Therefore, we believe that the regulation of NF-*κ*B is also a feasible way to reverse drug-resistant epilepsy, and this method also provides a clinical basis for the anti-inflammatory treatment of drug-resistant epilepsy.

## 5. Strategies for Reversing ABC Transporter-Mediated Drug Resistance

### 5.1. ABC Transporter Inhibitors

Dozens of P-gp inhibitors have been found and can be divided into 3 generations according to the selectivity of P-gp. The first generation includes cyclosporine A, verapamil, and similar drugs with poor selectivity. The second generation includes analogs of cyclosporine A, with stronger selective inhibition of P-gp, but these inhibitors can affect drug metabolism by affecting cytochrome P450. The third generation of blockers, such as LY335979 and OC144-093, can selectively block P-gp without affecting cell metabolism [44]. MRP inhibitors include probenecid, quinoline, cyclosporine, and similar drugs. Some ABC transporter inhibitors have been used in animal models and clinical experiments.

In animal experiments, Potschka et al. [45] found that the MRP antagonist probenecid increased the drug concentration of phenytoin sodium in the rat hippocampus in an amygdala kindling model, and probenecid also increased the anticonvulsive effects of phenytoin sodium on the kindling model. The drug concentration in the brains of rats lacking the MPR-2 gene was higher than that in the wild-type mice. The anticonvulsive effect of phenytoin sodium on this model was obviously stronger than that in wild-type mice. Potschka and Löscher [46] applied microdialysis technology to drug-resistant epilepsy animal models and found that the MRP1 inhibitor probenecid can improve the concentrations of carbamazepine and phenytoin sodium in the extracellular fluid of the rat cortex. Scism et al. [47] also found in rabbit models that, compared with an intravenous drip of valproic acid alone, a combined application of valproic acid and probenecid obviously increased the concentration of valproic acid in the brain fluid and parenchyma. The fact that the combination with probenecid obviously improved the drug concentration over that for a single application of valproic acid confirmed the clinical application prospects of MRP reversal agents.

On the basis of animal experiments, researchers attempted to use P-gp inhibitors for the clinical treatment of IE. Summers et al. [48] found that, after auxiliary application of the P-gp inhibitor verapamil in a 24-year-old female IE patient, the interval between complex partial seizures was prolonged and the control effects on epilepsy and patients' quality of life were greatly improved. The scholars Wang et al. [49] used the P-gp inhibitor flunarizine to treat IE patients. Fifty-four patients were randomly divided into a medication group and a control group. The medication group was divided into two groups: the P-gp positive group and the P-gp negative group. Immunohistochemistry was used to detect the P-gp expression in the medicated group. Flunarizine was administered, and the treatment was continued for 2 weeks. Six to eight weeks later, the clinical curative effects were evaluated. The treatment was considered effective when the attack number was reduced by 50%. P-gp-positive patients with valid results were rechecked for P-gp. The control group was administered placebo for synchronous observation. The results showed that 14 cases were valid among the 22 patients of the P-gp-positive group, 2 cases were valid among the 12 patients of the P-gp-negative group, and 4 cases were valid among the 20 patients of the control group. A total of 11 cases that were P-gp positive and had valid results completed the recheck of P-gp; in 10 of these cases, the P-gp expression was decreased by more than 50%. This finding indicated that the auxiliary treatment with flunarizine was effective, and its antiepileptic mechanism may be related to a reversal of the change in P-gp expression. Nicita et al. [50] found that add-on treatment with verapamil seems to have some effect in controlling seizures in patients with genetically determined Dravet syndrome (DS).

The clinical application of P-gp transporter inhibitors showed that the application of P-gp inhibitors also has certain risks. First, it is more difficult to inhibit P-gp in the BBB than in other areas; thus, larger doses of inhibitors are required to inhibit P-gp activity. These doses may cause complications due to the high concentration of drugs in nontargeted brain areas and peripheral tissues, thus causing adverse reactions. Second, ABC transporters in the BBB provide physiological protection of the central nervous system (CNS). P-gp inhibitors may increase the entry of various exogenous compounds into the CNS and generate accumulation toxicity. In addition, P-gp and the ABCC transporter have certain protective effects on brain tissue cell apoptosis [51, 52]; thus, the application of P-gp inhibitors may accelerate the apoptosis of brain cells.

### 5.2. Natural Drugs That Inhibit ABC Transporters

Due to the high toxicity of P-gp inhibitors, the role of the target is relatively simple; thus, P-gp inhibitors have not been used clinically. Additionally, traditional Chinese medicine (TCM) can provide comprehensive regulatory actions with few side effects, multiple targets, and multiple techniques; thus, TCM has the possibility of reversing MDR1 and P-gp expression. TCM treatments (such as treatments using* Ligusticum wallichii*,* Salvia miltiorrhiza*, peach seed, safflower,* Angelica sinensis*, radix paeoniae rubra, radix bupleuri, and* Alisma plantago-aquatica*) that are commonly used for tumor treatments perform functions that are similar to those of calcium channel antagonists. Furthermore, this type of TCM can be used for the auxiliary treatment of intractable epilepsy. Xie et al. [53] found that low, moderate, and high doses of Chaihu Shugan Decoction (particularly the high dose) can all reduce both the expression of P-gp in the hippocampus and temporal lobe cortex and the epileptiform discharge as revealed by electroencephalogram (EEG).

### 5.3. Other Treatment Strategies

Other treatment strategies include immune therapy and gene therapy (e.g., blocking MDR1 gene mutation and repairing gene defects), nanotechnology optimization, and multimechanism combination medication [54].

## 6. Summary

Although the IE drug resistance mechanism has not been fully elucidated, ABC transporters certainly play an important role in the multidrug-resistant process of IE. Aiming at altering drug efflux, future breakthrough points will be the development of drugs that are impervious to the discharge effect of ABC transporters by choosing the most suitable inhibitors of ABC transporters, the identification of AEDs with non-ABC transporters substrates, or changes in the dosage form. In conclusion, epilepsy drug resistance is a complicated process. Clarifying the mechanism can help to identify the basis of epilepsy. With in-depth studies of nerve physiology, molecular biology, and pharmacogenomics, the epilepsy drug resistance mechanism can be fully revealed, enabling the development of novel epilepsy medications.

## Figures and Tables

**Table 1:** Substrates and inhibitors of ABC transporters that are expressed at the blood brain barrier.

ABC transporter	Substrates	Inhibitors
P-glycoprotein (ABCB1)	Anticancer drugs, for example, doxorubicin, daunorubicin, vincristine, etoposide, teniposide, paclitaxel, and methotrexate	1st generation, for example, verapamil, cyclosporine A, quinidine, quinine, amiodarone, and detergents such as Cremophor EL
	Immunosuppressive agents, for example, cyclosporine A	2nd generation, for example, PSC-833 (valspodar), GF120918 (elacridar), VX-710 (biricodar), and dexverapamil
	Corticoids, for example, dexamethasone, hydrocortisone, corticosterone, cortisol, and aldosterone	3rd generation, for example, OC 144-093 (ONT-093), LY335979 (zosuquidar), XR9576 (tariquidar), R101933 (laniquidar), and GF120918
	Analgesics, for example, morphine HIV protease inhibitors, for example, amprenavir, indinavir, and saquinavir Cytokines, for example, IL-2, IL-4, and IFN-*γ* Antidiarrheal agents, for example, loperamide Anthelminthic agents, for example, ivermectin and abamectin Antigout agents, for example, colchicines Histamine H2-receptor antagonists, for example, cimetidine Calcium channel blockers, for example, verapamil Antiepileptic drugs, for example, phenytoin, carbamazepine, lamotrigine, phenobarbital, felbamate, gabapentin, and topiramate Antiemetics, for example, domperidone and ondansetron Cardiac glycosides, for example, digoxin Diagnostic (fluorescent) dyes, for example, rhodamine-123 Antidepressants, for example, amitriptyline, nortriptyline, doxepin, venlafaxine, and paroxetine Antibiotics, for example, erythromycin, valinomycin, tetracyclines, and fluoroquinolones	

MRP1 (ABCC1)	Anticancer drugs, for example, etoposide, teniposide, vincristine, doxorubicin, daunorubicin, and methotrexate; leukotriene C4 (LTC4), D4, and E4; various glutathione, glucuronide, and sulfate conjugates as well as unconjugated compounds (e.g., fluorescein)	Sulfinpyrazone, probenecid, MK571, LTC4, and some P-gp inhibitors (e.g., cyclosporine A, verapamil, and PSC 833)

MRP2 (ABCC2)	Similar to MRP1	Similar to MRP1

MRP3 (ABCC3)	Organic anion transporter with considerable overlap in drug substrates with MRP1 and MRP2	Classical organic anion transport inhibitors, for example, sulfinpyrazone, indomethacin, and probenecid

MRP4 (ABCC4)	Anticancer drugs, for example, methotrexate, 6-mercaptopurine, and thioguanine	

MRP5 (ABCC5)	cGMP, cAMP, 6-mercaptopurine, thioguanine, and fluorescein	Probenecid and phosphodiesterase inhibitors, for example, trequinsin or sildenafil

MRP6 (ABCC6)	BQ-123 (an anionic cyclopentapeptide and endothelin receptor antagonist)	

BCRP (ABCG2)	Several anticancer drugs; considerable overlap with P-gp, MRP1, and MRP2. Anthracyclines, mitoxantrone, bisantrene, the camptothecins topotecan and SN-38, prazosin, BNP1350, tomudex, and chlorine E6	GF120918 (also inhibits P-gp), fumitremorgin C (FTC), and FTC analogs, for example, Ko132, Ko134, CI1033, and estrone

From [13, 55–59].

**Table 2:** Immunohistochemical localization of P-gp in rat brains: comparison of different fixation and staining protocols.

Protocol	Pretreatment (ethanol/acetate)	Antibody	Control rats			Rats after kainate induced SE		
			Astrocytes	Endothelial cells	Neurons	Astrocytes	Endothelial cells	Neurons
#1 (acetone fixed cryostat sections of snap-frozen tissue)	−	C219	−	+	−	−	+	(+)
	−	mdr-(Ab-1)	−	+	−	−	+	+
	−	H241	−	(+)	−	−	+	(+)

#2 (paraformaldehyde-fixed cryosections)	−	C219	−	(+)	−	−	(+)	−
	−	mdr-(Ab-1)	(+)	(+)	−	+	(+)	−
	−	H241	−	(+)	−	−	(+)	−
	+	C219	−	+	−	−	+	−
	+	mdr-(Ab-1)	−	+	−	(+)	+	−
	+	H241	−	+	−	−	+	−

#3 (paraformaldehyde-fixed vibratome sections)	−	C219	−	−	−	+	−	−
	−	mdr-(Ab-1)	(+)	(+)	−	+	(+)	−
	−	H241	−	−	−	(+)	−	−
	+	C219	−	+	−	−	+	−
	+	mdr-(Ab-1)	−	+	−	(+)	+	−
	+	H241	−	+	−	−	+	−

Consistent immunostaining of P-gp in a specific cell type is indicated by +, weak staining by (+), and a lack of staining by −.
